# 

**Published:** 1891-03-07

**Authors:** 


### NOTICE TO CORRESPONDENTS.

All MS., Letters, Books for Review, and other matters intended for the Editor should be addressed The Editor, The Lodge, Porchester Square, London, W.

The Editor cannot undertake to return rejected M.S., even when accompanied by stamped directed envelope.

**Notice to Advertisers and Subscribers.**

All Advertisements, Orders for Copies of The Hospital, and Business Communications should be addressed to The Publisher, *not to the Editor*, The Hospital, 140, Strand, W.C.

The Cost of Subscription, post free, is as follows:—Per week, 1½d.; per quarter, 1s. 8d.; per half-year, 3s. 3d.; per annum, 6s. 6d. Foreign:—Per annum, 8s. 8d.; payable, in all cases, in advance.

### Scraps and Gleanings.

Influenza is said to have made ravages in Tokio, and many fatal cases have occurred.

A public mortuary for Hampstead was opened on Saturday in New End, near the workhouse.

During the last four years the Leeds workpeople have raised the sum of £18,452 for the Hospitals and Infirmary.

William Adlam, a labouring man, known as the Somerset centenarian, has just died at Taunton in his 107th year.

The Irish Distressed Ladies' Fund has received a donation of one hundred guineas from the Mercers' Company.

The balance-sheet for 1890 of the West Norfolk and Lynn Hospital shows an adverse balance of upwards of £290.

Willesden has adopted the Free Libraries Act. The figures were—For the adoption of the Act, 2,257; against, 1,070.

The subscriptions to the Burton Infirmary during 1890 amounted to £2,272 19s. 9d., which is more than in any previous year.

The receipts of the Dublin Hospital Sunday Fund amounted in 1890 to £4,188; the working expenses of the year being £273 3s. 3d.

On March 5th Professor C. Meymott Tidy began a course of three lectures on "Modern Chemistry in Relation to Sanitation," at the Royal Institute.

The entertainment at the Brompton Hospital on February 24th was given by Mrs. Frazer. The programme included vocal and instrumental music by the Fraser Institute.

The Duchess of Albany has consented to present the certificates awarded at the close of the course of lectures on domestic hygiene, to be given at the Sanitary Institute.

Mr. William Van Praagh gave a lecture on February 26th, at the Central London Throat, Nose, and Ear Hospital, on the pure oral system of teaching deaf mutes.

In consequence of the outbreak of small-pox in Belfast the Inspector-General of Constabulary has issued orders for the re-vaccination of the entire police force in that town.

The Indian medical examination was held at Burlington House on Feb. 9th. Fifty-nine candidates competed for twenty-one appointments, and fifty-seven were reported qualified.

The sixty-fifth annual report of the Suffolk General Hospital states that 460 in-patients and 1,235 out-patients were treated in 1890. The treasurer has a balance in hand of £20 12s. 6d.

A young man in Munich who has been treated with tuberculine—Dr. Koch's fluid—since December 2nd, has been dismissed as cured. He has gained eight pounds, and his general condition is excellent.

The Bishop of Winchester preached on Sunday morning at St. Mary Abbott's, Kensington, the offertory at the conclusion of the service being in aid of the funds of the Brompton Consumption Hospital.

A performance of "Othello" will be given at Langham Hall, on March 12th, in aid of the Paddington Green Children's Hospital. Tickets can be obtained from Dr. Whitefoord, 117, Albany Road, Regent's Park.

The report for 1889-90 of the Retford General Dispensary and Hospital shows that 27 cases were treated in the hospital, and 1,032 persons attended at the dispensary. The treasurer's account shows a balance in hand of £56 9s. 7½d.

The District Charitable Society of Calcutta intend to ask the Indian Government to appoint a Commission to inquire into the extent of the distress among the domiciled Europeans and Eurasians, and the best means of relieving and reducing it.

Several greenhouse workmen are said to be suffering from blood poisoning, through contact with a plant known as the Chinese primrose, the leaves of which have the property of irritating and even poisoning the skin of some people who touch it.

The annual report of the Lancaster Infirmary for 1890 shows that the deficit on the maintenance account has been reduced by the sum of £246. During the year 355 cases have been treated in the wards of the infirmary, and 3,439 as dispensary or dental cases.

A meeting was held on the 11th ult. with a view to assisting the work of the hospital for the dying. This unique hospital aims at supplying a much-felt want. For five years ten beds have been supported by Miss Davidson, but more beds are much needed.

A man called William Pratzman has died while practising the faith cure of the association calling themselves "Christian Scientists." Pratzman had been ill since December of typhoid pneumonia, but his relatives refused to call in a doctor, being converts to the so-called "faith cure."

Paignton Cottage Hospital and Provident Dispensary, which has been erected, furnished, and endowed by Messrs. Mortimer and Washington Singer, was formally opened on February 17th, by Mr. Studdy, C.C., in the presence of a large number of the friends of the donors.

The Governors of the Derbyshire Infirmary have decided to pull down the present building, and to erect new premises at a cost of £74,000. The Duke of Devonshire, as Lord Lieutenant of the County, has promised to call a county meeting for the purpose of raising the necessary funds.

According to regulations, all apothecaries engaged in the sale of Dr. Koch's lymph are required to return to Dr. Liebertz, at Berlin, whatever quantities of the fluid still remain unsold within six months of the time of purchase, when a fresh supply will be given in exchange without further cost.

The annual meeting of subscribers to, and friends of, the Exeter Dispensary was held on February 14th. Mr. W. Pring presided. The total number of patients admitted was 5,750. The receipts amounted to £1,955 7s. 7d., and the expenditure to £1,547 17s., showing a balance in favour of the institution of £407 1s. 7d.

The Marchioness of Londonderry visited the Children's Hospital, Belfast, on the 2nd inst., and expressed her satisfaction at the order and well-being observable throughout the building. Before leaving she announced her intention of presenting a cot to the Memorial Convalescent Home, which has recently been opened in connection with the hospital.

### The Student of Medicine.

[All communications for this Supplement should be addressed to The Editor of The Hospital, at 140, Strand, with the words, "Students' Column," written in the left hand top corner of the envelope.]

### LEADING ATHLETES.

Mr. Gardner, the renowned oarsman, whose portrait we give this week, was educated at Ascham School, Bournemouth, and afterwards at Rugby, but he did not long remain at Rugby, but finished his preliminary education with private tutors. At school he went in chiefly for gymnastics, but also for running and football. He went to Cambridge in 1883, and at once commenced rowing, winning his College sculls and trial eights in his first term; at the same time he got into his College football fifteen. In the Lent term, 1884, he rowed two in his College first boat, and in the May he rowed stroke, which place he occupied for six years, making during this time about twenty-four bumps. Again in 1884 he won the College sculls. In the following year he won the junior sculls at Bedford Regatta, and again the College sculls. In 1886, with S. Barnard as partner, he carried off the College pairs, the College sculls, and also the senior sculls and junior fours at the Bedford Regatta, and the Colquhoun sculls at Cambridge, and stroked the winning University trial eight. In 1887 for some time he rowed three in the University boat, but owing to illness he had to give way before the race. In the same year he carried off the diamond sculls at the Henley Regatta, but was beaten in the Wingfields at Putney. In the following December he again stroked the winning University trial eight. In 1888 he stroked the Cambridge University boat against Oxford, and won easily, but at Henley he lost the diamonds, and the Wingfield sculls at Putney. At the Bedford Regatta, however, he, with J. B. Peace as partner, won the gig pairs. In 1889 he again stroked the Cambridge crew to victory. With F. H. Cooke as partner, he won the College pairs. At Cambridge this year he, with S. D. Mutlebury as partner, won the University pairs, and also the goblets at Henley, but during this year he did not scull. In October, 1889, he entered St. George's Hospital, and played in the Rugby fifteen until his knee was injured. In this year he again stroked Cambridge, but at short notice, and, after a splendid race, was defeated. He stroked his Hospital four at the Inter-Hospital Challenge Fours and won. He was just defeated in the diamonds, but won the Wingfield sculls, or amateur sculling championship. Besides all these glories in the rowing world, Mr. Gardner has won prizes for running at various distances, long jump, and putting

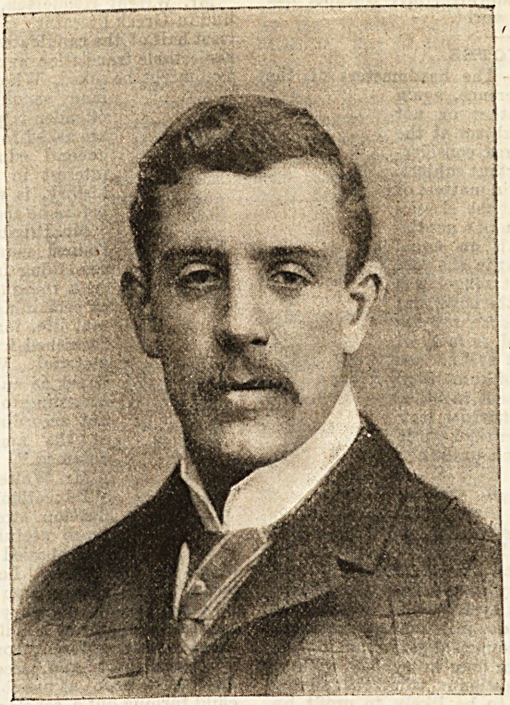

*From a photograph by Walery.*

Mr. J. C. GARDNER (St. George's).

### The Hospital Advertiser.

### THE STUDENT OF MEDICINE—(*continued*).

the weight; also for lawn tennis and fives, and last year, without training, he won the mile and half-mile at his Hospital sports. Since matriculating at Cambridge, Mr. Gardner has won over ninety prizes in the athletic world.

### EDITORIAL NOTES.

THE UNIVERSITIES AND GREEK. — The headmasters of the public schools, at their recent conference, again raised the important question whether or not Greek ought to be a compulsory subject at the Universities. Opinions were, of course, considerably divided on this, as on every important subject. Some men of considerable experience in matters of preliminary education maintained that the classics were the life essence of the education of a gentleman. Again, other authorities with an equal experience of teaching considered that in the vast majority of cases the Greek learnt at school was repulsive to the developing mind of the boys, and was, in fact, a great waste of patience for the teacher, and of time for the pupil; and, in fact, all these hours which under our present system are simply wasted as far as accumulation of knowledge is concerned, might be profitably spent in other subjects. To show how different authorities look upon this question, we cannot do better than quote from one or two letters on the subjects published in the *Times*.

Professor Edward A. Freeman, of Oxford, writes: "Is Greek, then, essential to those forms of liberal study which it is the business of an University to foster? I must confess that I cannot calmly argue the question. It is one of the things that one takes for granted. I can no more fancy what I should be without Greek than what I should be without eyes. And I believe that even those who take the other side do, in a sense, take it for granted also. Greek is allowed to be a council of perfection, only it cannot be required of everybody. Nor does everybody wish to require it of everybody. The only question is, whether it should be required of all who are supposed to be likely to profit by University studies. That is to say, are we to pitch our University standard so high that we can make Greek an essential part of it?"

Mr. Wright Henderson, of Oxford, says: "It is difficult to find in Greek literature a passage which would not 'pluck' at least half of the candidates, if anything like a creditable, even a respectable translation were to be exacted. Of course the question might be asked, Whose fault is this? It might be laid on many shoulders. I will only say that the worst offender is pass Greek itself. This poor stuff we are asked to preserve as an essential part of that general education which a youth with a real interest in mathematics, or natural science or history, is very properly called on to go through before he specialises."

Mr. Prigdin Teale, of Leeds, writes: "A well-trained man should enter upon his studies as something fresh, and with the delight of novelty as a thing to be greedily absorbed and retained, as pertaining no longer to 'ludus,' but to serious real life, for which his educational training has furnished him with tools, but not with the raw material. Viewed from such a point, the value of Greek as an educational subject may stand or fall according to its educational value pure and simple, not according to its quantity, still less in view of the amount that would be permanently retained."

Mr. Walter Wren, the well-known tutor, writes: "Education should do two things: (a) draw out, develop, strengthen the powers of the mind; (b) teach useful knowledge. Nothing can be taught and studied so well calculated to effect the first object as 'the Classics,' Latin and Greek. I believe Greek is even better for the purpose than Latin. Every boy and girl, too, should be taught Greek, and they should begin as early as possible, for two reasons. First, a child can learn a language whose written characters are different from our own, most easily when its mind is a clean sheet; and, secondly, it is desirable for teachers to find out as soon as possible whether there is any chance of a child turning out a scholar, for, if not, the study of both Greek and Latin should be dropped. One headmaster in writing on

### The Hospital Advertiser.

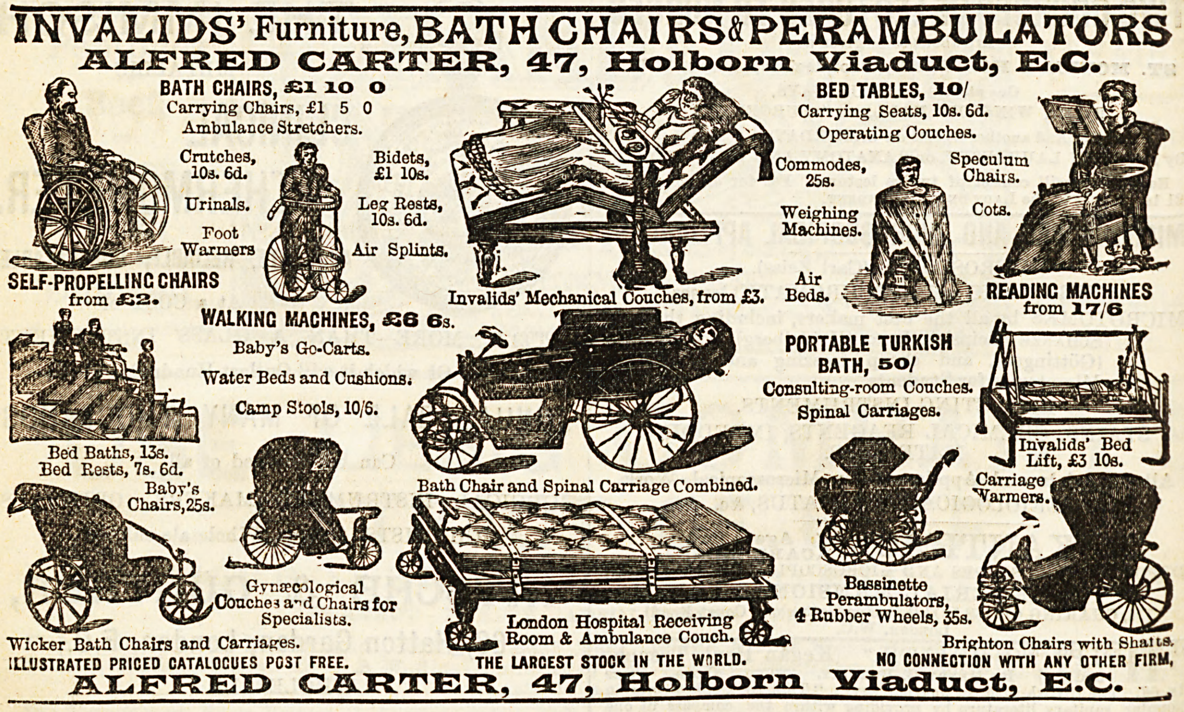

BRINSMEAD PIANOS

Keen's Mustard
THE OLDEST MANUFACTORY IN THE CITY OF LONDON.

**THE STUDENT OF MEDICINE**—(*continued*).

this subject, says: 'Even a slight knowledge of Greek acquired under compulsion may ripen into something more fruitful.' Certainly it may, but I never heard of a case where it did, or of anyone expressing a hope or belief that it would."

The remarks of Mr. Wren are certainly those which fit in most with our views of the subject. It is simply monstrous that a youth who intends to devote his life to some scientific profession should be compelled to waste years over a prehistoric subject, which Professor Huxley has aptly termed "the Latin superstition." How many of our greatest scientists never did any good at school? The explanation being that in most cases the orthodox classical subjects of a schoolboy's days were nauseating to their minds. It was only after the period of school life was passed that the opportunity for mental development occurred, and instead of being the stupids, as stamped by their teachers, they have proved themselves to possess the most original minds. Charles Darwin, in writing about his school days, says, "It was strictly classical," and then writes, "the school, as a means of education to me, was simply a blank." John Hunter was, perhaps, fortunate in that he never went through a classical education.

**APPOINTMENTS.**

At the London Hospital, Gabreil William Farmer, B.A., M.B., B.Ch. Oxon., M.R.C.S., L.R.C.P., has been appointed house physician. L. C. Everard Calthrop, L.R.C.P., M.R.C.S., resident accoucheur. At Guy's Hospital, J. H. Bryant, M.B., B.S. Lond., has been appointed assistant house physician. F. Wellford, M.B., B.C. Cantab., assistant house surgeon. T. G. Stevens, M.B., B.S. Lond., assistant house surgeon. At the Hospital for Consumption and Diseases of the Chest, Brompton, E. L. Rowse, L.R.C.P., M.R.C.S., has been appointed house physician. At University College Hospital, G. F. Blacker, M.R.C.S., L.R.C.P., has been appointed senior obstetric assistant. At the National Hospital for the Paralysed and Epileptic, Queen's Square, Bloomsbury, C. A. Ballance, F.R.C.S. Eng., has been appointed surgeon. At the Liverpool Infirmary for Children, William Ker Bell, M.R.C.S., L.R.C.P., has been appointed assistant house surgeon. At the East Suffolk and Ipswich Hospital, A. C. Coles, M.B., C.M., B.Sc., has been appointed assistant house surgeon. At the Kent and Canterbury Hospital, Arthur C. Elliman, M.R.C.S., L.R.C.P. Lond., has been appointed assistant house surgeon. At Owens College, Manchester, T. N. Kelynack, M.B., Ch.B. Vict., has been appointed junior demonstrator in pathology. Robert B. Wild, M.D. Lond., B.Sc., M.R.C.S., has been appointed senior demonstrator of materia medica and therapeutics. At the Dorset County Hospital, Dorchester, B. P. Viret, M.R.C.S. Eng., L.R.C.P. Lond., has been appointed house surgeon. At the Central London Sick Asylum, Cleveland Street, W., Arthur G. Welsford, M.B., B.C. Cantab., has been appointed assistant medical officer. At the Royal Portsmouth, Portsea, and Gosport Hospital John Watson, M.B. Durh., F.R.C.S. Edin., has been appointed senior house surgeon. At the Hobart General Hospital, Tasmania, Kenneth Maxwell, M.B., C.M. Edin., has been appointed assistant house surgeon. Alfred J. Turner, M.D. Lond., M.R.C.S., has been appointed house surgeon.

**VACANCIES.**

The following vacancies are announced this week, the amount of salary in each case being given after the name of the institution:

**Resident Medical Officers.**

Addenbrooke's Hospital, Cambridge, House Physician—Salary £65, and board.
Chelsea Hospital for Women, Fulham Road, S.W., Medical Officer—Salary £80, &c.
City Asylum, Birmingham, Clinical assistant.
General Hospital, Birmingham, Resident Medical Officer—Salary £130, &c.
Liverpool Infirmary for Children, Myrtle Street, House Surgeon—Salary £85, and board, &c.
Manchester Royal Infirmary, Resident Medical Officer to the Convalescent Hospital at Cheadle—Salary £150.
National Dental Hospital, Great Portland Street, W., House Surgeon—Salary £50.
National Hospital for Consumption, Ventnor, Dispenser—Salary £60, &c.
North-Eastern Hospital for Children, Hackney Road, N.E., Junior House Surgeon for nine months—Salary £5 a month.
Rotherham Hospital, Assistant House Surgeon.
Royal Bath Hospital and Rawson Convalescent Home, Harrogate, House Surgeon and Secretary—Salary £80, &c.
St. Peter's Hospital for Stone and Urinary Diseases, Henrietta Street, Covent Garden, House Surgeon—Honorarium 25 guineas, &c.
St. John's Hospital for Diseases of the Skin, Leicester Square, W.C., House Surgeon.
West Ham Union, Assistant Medical Officer—Salary £100, &c.

### THE STUDENT OF MEDICINE—(*continued*).

### Non-Resident Medical Officers.

St. George's and St. James's Dispensary, King Street, Regent Street, Surgeon.
Dental Hospital of London and School of Dental Surgery, Four Demonstrators—Honorarium £50.
Hospital for Women, Soho Square, W., Clinical Assistants.
Hull Royal Infirmary, Four Honorary Assistant Surgeons; also Ophthalmic Surgeon.
Miller Hospital and Royal Kent Dispensary, Greenwich, S.E., Physician.
Skirlaugh Union, Medical Officer and Public Vaccinator for the district of Sproatley—Salary £20, exclusive of fees.

### PASS LISTS.

#### University of London.

INTERMEDIATE EXAMINATION IN MEDICINE, January, 1891.

Entire Examination.—*First Division*: Henry George Felkin, London Hospital; James Ryland Hickinbotham, Queen's College, Birmingham; Charles Paine, St. Mary's Hospital; Frederick John Poynton, St. Mary's, Hospital; Herbert Campbell Thomson, Middlesex Hospital. Herbert James Walton, St. Bartholomew's Hospital. *Second Division*: Christopher Addison, St. Bartholomew's Hospital; Martin Ashley' University College; John Aaron Berlyn, Queen's College, Birmingham; Ramon Horace Castellote, University College; Robert Francis Chance, St. Thomas's Hospital; Dudley Willis Collings, St. Bartholomew's Hospital; Harry Guy Dain, Queen's College, Birmingham; Herbert Fox, Glasgow Royal Infirmary and Middlesex Hospital; Bertram Harold Kingsford, St. Thomas's Hospital; Robert Casement Kirkby, Guy's Hospital; Ernest Graham G. Little, St. George's Hospital; Thomas Davys Manning, Guy's Hospital; Mary Ellen Rye, London School of Medicine for Women; Samuel Robert Schofield, University College; Walter Scott H. Sequeira, London Hospital; William Shears, St. Bartholomew's Hospital; George Nathan Oscroft Slater, Sheffield, and St. Bartholomew's Hospital; Kirsop B. James Vickers, St. Thomas's Hospital.

Excluding Physiology.—*First Division*: Francis Parris Piper, St. Mary's Hospital; Courtenay Mausel Rhodes, St. Mary's Hospital; Edwin Sly, King's College. *Second Division*: Evelyn Lancelot Adams, Guy's Hospital; Arthur Percy Allan, Guy's Hospital; Hugh Kennedy Birley, Owens College; Arthur James Edge, St. Bartholomew's Hospital; Percy Stanhope Eves, University College; Charles Frederick Gross, King's College; George Frederick Murrell, St. Bartholomew's Hospital.

Physiology Only.—*First Division*: Leonard Arthur Bidwell, St. Thomas's Hospital; Alfred George Jones, Middlesex Hospital. *Second Division*: Robert Turle Bakewell, University College; Lullum Wood Bathurst, St. Bartholomew's Hospital; Alfred Herbert Card, Guy's Hospital; William Henry Jewell, Guy's Hospital; George Martyn, King's College; Alfred Hugh Minton, King's College; Albert Paling, Middlesex Hospital; William Charles Cunningham Park, Guy's Hospital; Reginald Smith, Owens College.

### ATHLETICS.

The Dental Hospital Athletic Club.—The second annual concert of this club took place on Saturday, January 31st, in the International Hall of the Café Monico. Mr. Woodhouse Braine was in the chair. The programme was well varied, comic songs being the chief items. Most of the performers were members of the Dental Hospital Musical Society, but several professionals were also kindly present. The hall was well filled and very noisy, so much so that many of the solos had to be omitted, in some cases it being impossible to hear the singer's voice on account of the rowdyism of a few present in the back part of the hall. The ventriloquist, Mr. J. Eagles, was very good and excellently received, his range of voice being very wonderful. One of the successes of the evening was Mr. D. Hepburn's humorous song called "Transcen-dental Ditty," which was very well rendered. So also Mr. C. E. Walbourne's "Tootsie Sloper" and Mr. Bowles' comic song were excellent. Mention must be made of the banjo duet by Messrs. O. C. Sibley and Waller, who played "The Grand Avenue March" with much force. "The Heart of a Sailor" was well sung by Mr. Coysh, so also "Tell Her I Love Her So" by Mr. P. Leigh. Other gentlemen who took part in the performance were Mr. A. May, Mr. H. E. Bullen, who sang several times and was very popular, Mr. E. Trydell, Mr. R. Hershell, and Mr. T. Breeze. Unfortunately several of the higher toned pieces had to be omitted from the programme, such as part songs by the Musical Society, Mr. Wheatley's songs, a duet, violoncello and piano, by Messrs. Watts and Bullen, so also the song "'Mona," by Mr. P. Arles. We recommend the Dentals in the future to be much stricter with regard to the distribution of tickets for their concerts, and to see that none are admitted without them in order that a concert which looked so well on paper should not be spoilt by the rowdyness of a few outsiders who happened by some means to gain admission.

#### Football.

Inter-Hospital Challenge Cup (Second Round).—On Tuesday, February 10, the match in the second round of the challenge cup was played off between Guy's and Charing Cross at Richmond. The former were decidedly the stronger team, and won by three goals and a try to nothing. At the Athletic Ground, Richmond, on February 13, St. Thomas's (holders) and St. George's played their tie in the Hospitals cup competition. The former proved to be much the stronger side. Within eight minutes of the start Ogilvie dropped a goal, and by half time St. Thomas's lead had increased to three goals and a try. After changing ends, the holders gained another goal and five tries, and thus won by four goals and six tries to nothing. In the pavilion afterwards, the committee drew the penultimate round as follows: St. Thomas's v. Middlesex; Guy's, a bye.

